# Synthesis of nanoscale zero-valent iron by one-pot route and study of its potential in passivating coexistent heavy metal anions and cations in soil

**DOI:** 10.1039/d5ra02186c

**Published:** 2025-08-29

**Authors:** Xinzhe Zhang, Zhihao Yang, Xiaoya Bi, Yi Zhang, Yage Liu, Yanbao Zhao, Jianping Chen, Laigui Yu, Xueyan Zou

**Affiliations:** a Engineering Research Center for Nanomaterials, The First Affiliated Hospital, The Academy for Advanced Interdisciplinary Studies, College of Chemistry and Molecular Sciences, Henan University Zhengzhou 450046 China jp_chen@henu.edu.cn 422281732@qq.com zouxueyan@henu.edu.cn; b State Environmental Protection Key Laboratory of Soil Environmental Management and Pollution Control, Nanjing Institute of Environmental Sciences, Ministry of Ecology and Environment of China Nanjing 210042 China; c Food and Pharmacy College, Xuchang University Xuchang 461000 China

## Abstract

Nanoscale zero-valent iron (nZVI) was synthesized by a one-pot liquid-phase chemical method in the presence of FeSO_4_ as the iron source and NaBH_4_ as the reducing agent. The synthesized nZVI was characterized by scanning electron microscopy, X-ray diffraction, energy dispersive spectrometry, and Fourier transform infrared spectroscopy. Its ability to passivate Pb^2+^, Cd^2+^, and AsO_4_^3−^ in soils was evaluated by inductively coupled plasma-atomic emission spectroscopy, and the passivation mechanism was explored based on adsorption thermodynamics and kinetics simulations. It was found that nZVI is spherical in shape with a diameter of 60–80 nm and exhibited a satisfactory magnetic response, favoring facile recycling under a magnetic field, which could be directly applied to passivate Pb^2+^, Cd^2+^, and AsO_4_^3−^ in contaminated soils. The passivation ability for Pb^2+^, Cd^2+^, and AsO_4_^3−^ depended on the drying conditions and the dosage of NaBH_4_. Notably, nZVI prepared with 4 g of NaBH_4_ under vacuum drying exhibited the strongest passivation ability. The adsorption of the tested heavy metals by nZVI conformed to the Langmuir isotherm model, and the correlation coefficients were 0.99 (Pb), 0.99 (Cd), and 0.93 (As), which indicated saturated monolayer adsorption. The corresponding maximum saturated adsorption amounts were 117.65 mg g^−1^ (Pb), 45.45 mg g^−1^ (Cd), and 6.82 mg g^−1^ (As), respectively. Additionally, the adsorption by nZVI of the heavy metal ions under investigation followed the pseudo-second-order kinetic equation, referring to chemical adsorption, and the chemisorption percentages for Pb^2+^, Cd^2+^, and AsO_4_^3−^ were 93.0%, 74.8%, and 32.9%, respectively. This could account for the difference in the adsorption capacity of nZVI for the tested heavy metal ions. Moreover, 19 consecutive days of desorption experiments demonstrated that nZVI/M (M represents Pb, Cd, and As; *i.e.*, Pb^2+^, Cd^2+^, and AsO_4_^3−^) possessed strong stability. Our data indicate that nZVI has the potential to be an excellent nano-adsorbent with good passivation performance for the rapid and efficient passivation of Pb^2+^, Cd^2+^, and AsO_4_^3−^ in multi anion–cation co-contaminated soils.

## Abstract 2

Nanoscale zero-valent iron (nZVI) was synthesized by a one-pot liquid-phase chemical method in the presence of FeSO_4_ as the iron source and NaBH_4_ as the reducing agent.

## Graphical abstract

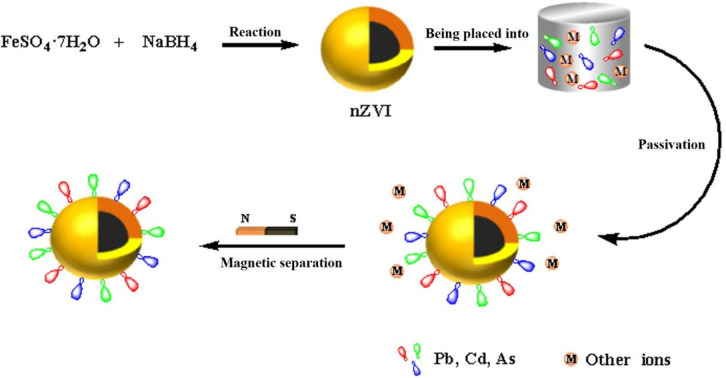

## 1 Introduction

With the rapid growth in mining, smelting, electroplating, chemical, electronics industries, untreated wastewater is increasingly being released, damaging ecosystems and the environment.^1,2^ Among a variety of pollutants, heavy metals are the primary ones that endanger the ecosystems, food safety, and human health.^3–5^ Untreated discharge of heavy metals and their compounds in gas, water, or solid waste harms ecosystems and the environment. The executive report of the United Nations Environment Programme lists Hg, Pb, Cd, As, Cr, Cu, V, Mo, Sn, Co, Ni, Sb, and Se as the most harmful elements.^5,6^ The characteristics of heavy metal pollution are high toxicity, easy enrichment, difficult degradation, irreversibility, strong concealment, and a wide scope. Heavy metals easily enter the food chain and then the human body to induce various diseases. For example, excessive amounts of Cd can cause osteoporosis, kidney damage, and even cancer; excessive Pb can severely damage the blood, kidneys, immune system, and other organs as well as the respiratory system to cause asthma and kidney diseases.^7–10^

AsO_4_^3−^is a major environmental hazard due to its carcinogenicity and mobility in the environment. Long-term exposure to arsenate is linked to cancer and organ damage. AsO_4_^3−^ contamination remains prevalent despite regulatory controls.^11^ To curb the expansion of heavy metal pollution, China has formulated a series of initiatives, including the “twelfth five-year plan for the comprehensive prevention and control of heavy metal pollution”, focusing on Pb, Cd, and As.^12,13^ These metals are particularly emphasized in national environmental policies because they are recognized for their severe environmental and health impacts, including neurotoxicity, carcinogenicity, and long-term soil and water contamination.^14^

At present, the remediation methods for heavy metal-contaminated soil mainly include physical, chemical, and biological strategies for removal. Physical methods, such as thermal treatment and guest soil method, are costly and greatly impact the soil environment;^15,16^ chemical methods, such as leaching, are prone to secondary pollution;^17^ and bioremediation is inexpensive but results in a long repair cycle and poor removal efficiency.^18^ Therefore, it is essential to develop effective methods and new remediation materials so as to control heavy metal pollution.

With the development of nanotechnology, nano-remediation agents, such as carbon-based, silica loaded, and magnetic nanomaterials, have been widely used for heavy metal-contaminated soil remediation.^19–22^ For example, Matos *et al.* used multi-walled carbon nanotubes to passivate the heavy metals Pb, Cu, Ni, and Zn in soil.^23^ Feng *et al.* used electrospun nanofiber membranes (ENFM) in water treatment for the adsorption of heavy metals, discussed the adsorption performance of different types of ENFMs, and emphasized the importance of improving their stability and reusability.^24^ Mohamadiun *et al.* adopted polyacrylic acid-stabilized nano-iron oxide to remove Cd from soil.^25^

Magnetic nano zero-valent iron (denoted as nZVI) is inexpensive, readily available, highly efficient for remediation, and environmentally friendly. Compared with ordinary zero-valent iron, nZVI exerts a unique small size effect and surface effect, and it also possesses a large specific surface area-energy, and therefore, the latter could exhibit higher reactivity and passivation efficiency.^26,27^ Additionally, the unique core–shell structure of nZVI, consisting of a zero-valent iron core and an Fe(ii)/Fe(iii) oxide shell,^28–31^ makes is water-soluble and magnetic, enabling rapid separation and minimizing secondary pollution. Therefore, nZVI might be of significance for remediating heavy metal-contaminated soil and/or water. What should be emphasized is that the currently available methods for fabricating nZVI are unsatisfactory because of their long reaction times, complicated reaction procedures, and relatively low yield.^32–36^ Additionally, issues such as rapid aggregation, surface oxidation, and poor dispersion in environmental matrices have hindered the practical application of nZVI in soil and water remediation.^37^

To address those issues, herein, we adopted a facile liquid-phase chemical method to synthesize nZVI in the presence of ferrous sulfate heptahydrate and sodium borohydride as the iron source and reductant, respectively. The as-prepared nZVI can be directly applied to remove the heavy metals Pb, Cd, and As from soil, especially under the action of an external magnetic field. This article describes the preparation and characterization of nZVI, as well as the evaluation of its passivation performance towards the heavy metals under investigation. It also discusses the passivation mechanism of nZVI based on adsorption–desorption experiments, as well as adsorption thermodynamics and kinetics simulations.

## 2 Materials and methods

### 2.1 Materials

Ferrous sulfate heptahydrate (FeSO_4_·7H_2_O, 99.0–101.0%) was obtained from the Sinopod Chemical Reagent Company Limited; sodium borohydride (NaHB_4_, 97%) was purchased from Luoyang Haohua Chemical Reagent Company Limited. Diethylene triamine pentaacetic acid (DTPA, 99%) and anhydrous ethanol (C_2_H_5_OH, 99.7%) were purchased from Anhui Kuer Biological Engineering Company Limited and Anhui Ante Food Company Limited, respectively.

### 2.2 Preparation of nZVI under different drying conditions

First, 4.45 g of FeSO_4_·7H_2_O and 70 mL of deionized water were successively added to a 200 mL flask under mechanical stirring until complete dissolution occurred. Into the resultant solution, 10 mL of NaBH_4_ solution was added dropwise (0.20 g of NaBH_4_ dissolved in 10 mL of deionized water) at a rate of 1 drop per s. During the addition of NaBH_4_ solution, the mixed solution gradually turned black and produced abundant bubbles, accompanied by a release of heat. The reaction stopped when bubble production ceased, and then, the product was washed with anhydrous ethanol and deionized water, three times each. The as-washed product was magnetically separated and dried at 60 °C for 48 h under ordinary conditions (atmospheric environment) or *in vacuo*. The as-obtained products, named nZVI-V and nZVI-O, were sealed in plastic bags and stored.

### 2.3 Preparation of nZVI with different amounts of NaBH_4_

FeSO_4_ solution (4.45 g of FeSO_4_·7H_2_O dissolved in 60 mL of deionized water) was prepared eight times under full mechanical stirring. Then, 20 mL of NaBH_4_ solution with different concentrations (1.00 g, 2.00 g, 3.00 g, 4.00 g, 5.00 g, 6.00 g, 7.00 g, and 8.00 g of NaBH_4_ were respectively dissolved in 20 mL of deionized water) was slowly (1 drop per s) added to the FeSO_4_ solutions. The reaction was terminated when bubble generation ceased, followed by washing with anhydrous ethanol and water, three times each. The as-washed product was magnetically separated and dried at 60 °C *in vacuo* for 48 h to obtain the target products named nZVI-1g, nZVI-2g, nZVI-3g, nZVI-4g, nZVI-5g, nZVI-6g, nZVI-7g, and nZVI-8g, which were sealed and stored in plastic bags.

### 2.4 Influence of the quantity of nZVI on adsorption capacity

To determine the influence of nZVI, 5.00 g of heavy metal-contaminated soil samples was added to a leach flask, followed by the addition of 1.00 g of nZVI-8g. Into the as-obtained mixed solution, 25 mL of diethylene triamine pentaacetic acid (DTPA) leaching solution was added (containing 0.005 mol per L DTPA, 0.01 mol per L CaCl_2_, and 0.1 mol per L triethylamine (TEA), pH 7.30). The flask then underwent 2 h of mixing on a orbital shaker-incubator (180 r·min^−1^, 25 °C). DTPA is a widely used chelating agent in environmental science, particularly in soil remediation, to extract metal ions. It forms stable complexes with metal cations, effectively separating them from the soil matrix. This chelation process is particularly beneficial for the extraction of heavy metals such as Pb^2+^, Cd^2+^, and AsO_4_^3−^, which are present in the contaminated soil samples. Upon completion of shaking, the solution was maintained undisturbed at room temperature for 1 h, and the supernatant was filtered and then used for inductively coupled plasma-atomic emission spectroscopy (ICP-AES) analysis (Optima 2100DV, USA). The experiment with the addition of 4.00 g of sample nZVI-8g was conducted in the same manner.

### 2.5 Effects of the quantity of NaBH_4_ and drying conditions on adsorption capacity

For this experiment, six samples were prepared, each containing 5.00 g of heavy metal-contaminated soil in an extraction bottle, and five samples were treated with 0.15 g of nZVI variants (nZVI-1g, nZVI-4g, nZVI-8g, nZVI-V, and nZVI-O). The sixth sample was prepared without the addition of nZVI to serve as a blank control to account for the potential background desorption of heavy metals induced by the DTPA solution. The extractable metal concentrations from this control were used as a reference baseline for evaluating the net immobilization efficiency of each nZVI sample.

### 2.6 Scaled-up preparation of nZVI

For the creation of a large quantity of nZVI, 17.80 g of FeSO_4_·7H_2_O and 300 mL of deionized water were successively placed in a 1000 mL flask and mechanically stirred to achieve complete dissolution. Subsequently, 100 mL of NaBH_4_ solution (20 g of NaBH_4_ dissolved in 100 mL of deionized water) was dropped into the above solution at a rate of 1 drop per s. The reaction was stopped when bubble production ceased. The product was washed with anhydrous ethanol and water, three times for each, followed by magnetic separation and drying at 60 °C for 48 h *in vacuo* to afford sample nZVI-bulk.

### 2.7 Adsorption–desorption behaviour

To study adsorption and desorption, 0.15 g of sample nZVI-bulk was placed into a 50 mL centrifuge tube, and 30 mL of Pb^2+^ solution (1000 mg L^−1^) was subsequently added. The mixture was shaken at 25 °C for 24 h in a constant temperature shaker. The supernatant was collected *via* magnetic separation to obtain nZVI-left. Then, 10 mL of distilled water was added to the precipitate (nZVI/M; M = Pb, Cd, and As), which was ultrasonically washed for 3 min and subsequently magnetically separated to afford the supernatant (recorded as nZVI-0) containing physically adsorbed ions. The precipitate was then subjected to ultrasonic dispersion in 10 mL of distilled water, shaken at 25 °C for 4 h, with shaking intervals every 2 h, followed by magnetic separation to obtain sample nZVI-1. The concentration of the ion desorbed on the first day was determined by ICP-AES. Those concentrations of the desorbed ions during a test duration of 2–19 days were measured using the same modality (the corresponding samples, the supernatants, are denoted as nZVI-2, nZVI-3, nZVI-4 up to nZVI-19). Similarly, the experiments for Cd^2+^ and AsO_4_^3−^ were carried out in the same manner.

### 2.8 Adsorption thermodynamics

To examine adsorption thermodynamics, 0.03 g, 0.06 g, 0.09 g, 0.12 g, and 0.15 g of sample nZVI-bulk and 30 mL of Pb^2+^ solution (1000 mg L^−1^) were added to 50 mL centrifuge tubes and oscillated at 25 °C for 24 h in a constant temperature shaker to achieve saturation adsorption, followed by magnetic separation to afford the supernatants for ICP-AES quantitative detection. The experiments for the Cd^2+^ and AsO_4_^3−^ solutions were performed in the same mode except that the dosage of sample nZVI-bulk was selected as 0.05 g, 0.06 g, 0.07 g, 0.08 g and 0.09 g, or 0.05 g, 0.10 g, 0.15 g, 0.20 g and 0.25 g.

### 2.9 Adsorption dynamics

To examine adsorption dynamics, 0.15 g of nZVI-bulk and 30 mL of Pb^2+^ solution (1000 mg L^−1^) or AsO_4_^3−^ solution (1000 mg L^−1^) were added to a 50 mL centrifuge tube to obtain the to-be-tested solution; nine of the same solutions were formulated and oscillated with a constant temperature shaker for 4 min, 8 min, 12 min, 16 min, 20 min, 24 min, 28 min, 32 min, and 24 h (25 °C, 180 r·min^−1^). Upon completion of shaking, the supernatants were immediately magnetically separated and used for ICP-AES quantitative detection. The experiments for the Cd^2+^ solution were conducted in the same mode except for passivation time (8 min, 16 min, 24 min, 32 min, 40 min, 48 min, 56 min, 64 min, and 24 h). All experiments were carried out under standardized conditions. While not labeled as triplicates, multiple parameter sets (*e.g.*, different NaBH_4_ dosages, drying conditions, and metal concentrations) served as internal technical parallels to ensure data reliability.

### 2.10 Reusability evaluation of nZVI

To assess the recyclability of the synthesized nZVI, repeated immobilization experiments were performed over three consecutive reuse cycles. Initially, 0.15 g of nZVI-bulk was used to treat 5.00 g of contaminated soil in a 50 mL centrifuge tube with the addition of 25 mL of DTPA extraction solution (0.005 mol per L DTPA, 0.01 mol per L CaCl_2_, 0.10 mol per L TEA, pH 7.3). The mixture was shaken at 180 r·min^−1^ for 2 h at 25 °C and then allowed to stand for 1 h. The supernatant was filtered and analyzed by ICP-AES to determine the residual concentrations of Pb^2+^, Cd^2+^, and AsO_4_^3−^. The nZVI remaining after magnetic separation was rinsed with deionized water, air-dried, and reused under the same conditions for three subsequent cycles. The immobilization rates were calculated for each round and labeled as nZVI-0 (fresh), nZVI-1, nZVI-2, and nZVI-3.

### 2.11 Comparative study of the different remediation materials

First, 5.00 g of contaminated soil and 0.15 g of respective remediation material (nZVI, lime, phosphate, or bentonite) were added to 50 mL centrifuge tubes to form four parallel groups, corresponding to an amendment dosage of 3% (w/w). Subsequently, 25.00 mL of DTPA extraction solution (0.005 mol per L DTPA, 0.01 mol per L CaCl_2_, and 0.10 mol per L TEA, pH 7.3) was added to each tube. The mixtures were oscillated at 25 °C for 2 h in a constant temperature shaker (180 r·min^−1^), followed by standing for 1 h at room temperature. The supernatants were then collected by filtration through 0.45 μm membranes and analyzed by ICP-AES to determine the concentrations of Pb^2+^, Cd^2+^, and AsO_4_^3−^. Immobilization rates were calculated relative to the untreated control group.

### 2.12 Analysis methods

The morphology, microstructure, and chemical features of the as-synthesized products were characterized by Fourier transform infrared (FT-IR) spectroscopy (Nicolet 6700, USA), X-ray single crystal diffractometry (XRD) (X'PERT Philips, Netherlands), and scanning electron microscopy (SEM) (JSM-5600, Japan). A high precision CNC shaking machine (ATS-03 M2R, Shanghai Kanxin Instrument Company Limited, China) was deployed to evaluate the adsorption performance of the as-prepared nano-adsorbents, and ICP-AES analysis was conducted to determine the concentration of the adsorbed heavy metals.

## 3 Results and discussion

### 3.1 Structural characterization of nZVI

**Fig. 1:**
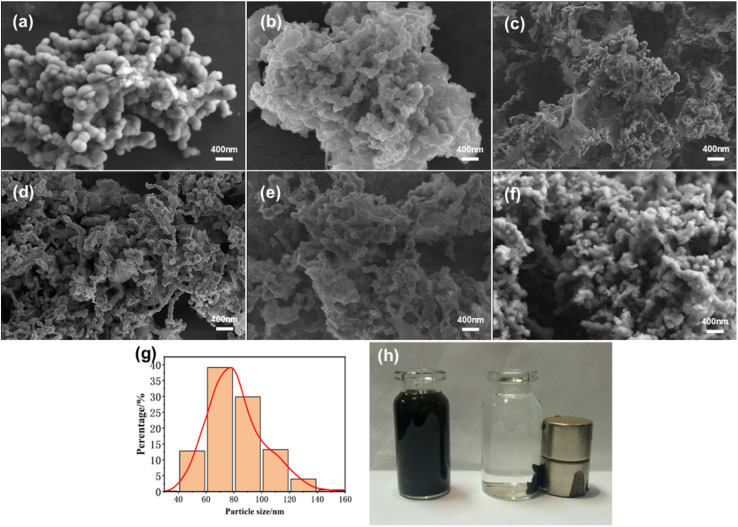
SEM images of nZVI synthesized with different amounts of NaBH_4_ (a: 1 g; b: 4 g; c: 5 g; d: 6 g; e: 7 g; and f: 8 g), as well as (g) particle size distribution and (h) a photograph showing the magnetic response of nZVI.

NaBH_4_, a strong reductant, not only reduces Fe^2+^ to Fe but also easily reacts with H_2_O to produce H_2_, and both are competitive reactions. To determine the optimal dosage of NaBH_4_, we prepared nZVI with different amounts of NaBH_4_. The SEM images in Fig. 1a–f show that the as-synthesized nZVI products exhibit relatively rough surfaces and irregular spherical shapes. With increasing NaBH_4_ dosage, the as-obtained nZVI particles gradually decrease in size (60–80 nm) and tend to agglomerate.^38^ This agglomeration is driven by the hydrogen generated during the reaction, which promotes the combination of nZVI particles into larger aggregates^39,40^ .

The photograph in Fig. 1h illustrates the magnetic response of nZVI, characterized by even dispersion that occurred in water, and quick separation that proceeded under the action of the external magnetic field. This characteristic indicates that the dispersion of nZVI in water is satisfactory, with strong magnetic responsiveness that can be applied in practical recovery processes.

**Fig. 2:**
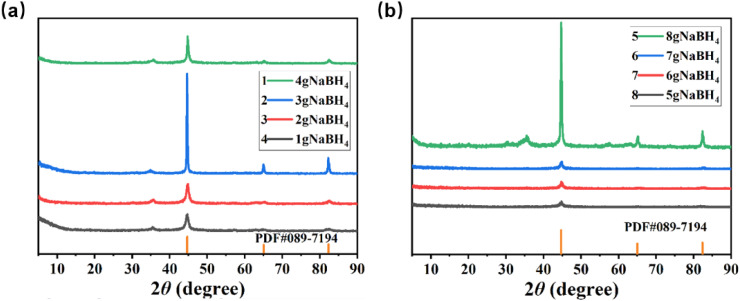
XRD patterns of nZVI synthesized with different amounts of NaBH_4_: (a) 1–4 g NaBH_4_, (b) 5–8 g NaBH_4_. The orange markers indicate the reference diffraction peaks of iron oxides (PDF #089-7194).

Fig. 2 shows the XRD patterns of nZVI synthesized with different amounts of NaBH_4_. The diffraction peaks at 2*θ* of 44.7°, 65.0°, and 82.3° correspond to the (110), (200), and (211) crystal facets of nZVI, referring to cubic metallic iron (PDF 89-7194), which are overlaid in Fig. 2 (orange markers) as references to assist in identifying the surface oxidation phases of nZVI. These peaks are sharp, which indicates the satisfactory crystallinity of the as-prepared nZVI products. In addition, the XRD signals at 2*θ* of 43.5°, 56.2°, 64.0°, 75.5°, and 80.6° were assigned to the (202), (211), (300), (220), and (312) crystal facets of cubic iron oxides (PDF #73-0603), respectively, suggesting that there is a thin layer of iron oxide on the surface of nZVI. This observation is in agreement with previous reports stating that nZVI often forms a thin oxide layer upon exposure to air or during synthesis.^41^

### 3.2 Evaluation of passivation performance

**Fig. 3:**
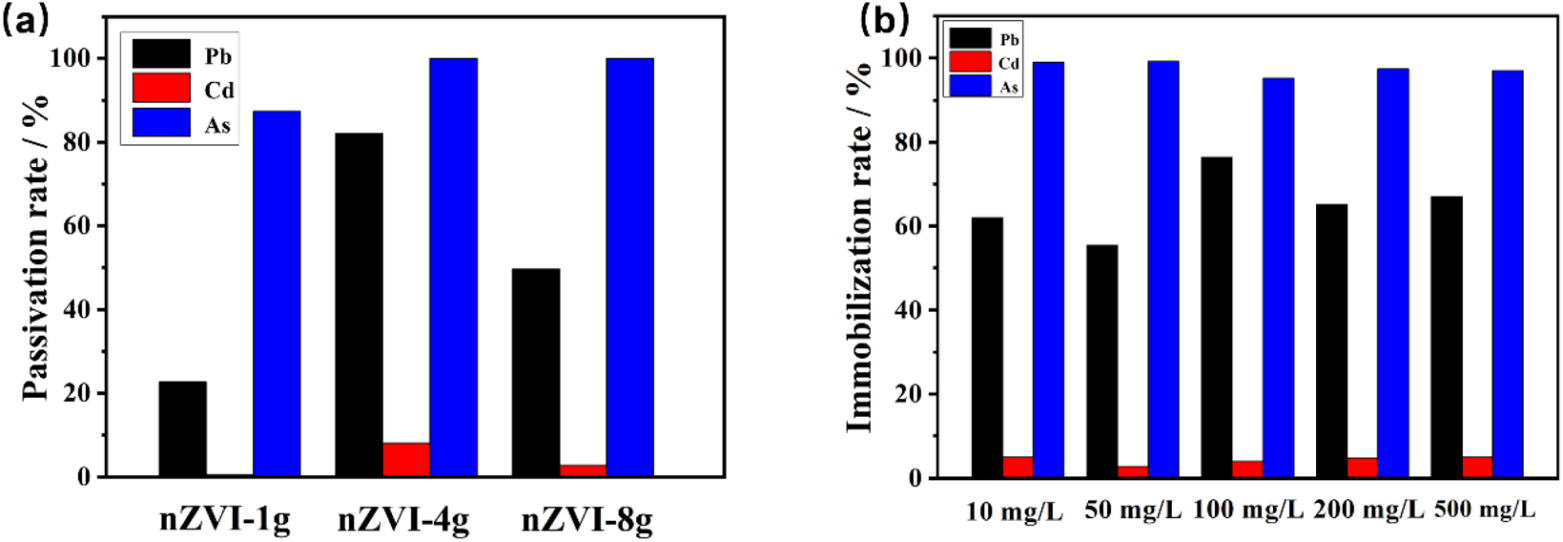
The relationship between the passivation rate of the tested heavy metals and the dosage of NaBH_4_. (a) The passivation rates for different dosages of nZVI (1 g, 4 g, and 8 g) in the presence of Pb, Cd, and As. (b) The immobilization rates for various initial concentrations of heavy metals (Pb, Cd, As) ranging from 10 mg L^−1^ to 500 mg L^−1^.

We used AsO_4_^3−^, Pb^2+^, and Cd^2+^ in soil to investigate the passivation efficiency of nZVI synthesized with 1 g, 4 g, and 8 g of NaBH_4_. Fig. 3 shows the relationship between the passivation rate of the tested heavy metals and the dosage of NaBH_4_. The passivation agents nZVI-1g, nZVI-4g, and nZVI-8g provided passivation rates of 87.41%, 99.99%, and 99.99% toward AsO_4_^3−^, exhibiting good passivation ability. The highest passivation rates of 82.09%, 8.02%, and 100% for Pb^2+^, Cd^2+^, and AsO_4_^3−^, respectively, were obtained for nZVI-4g. The comparatively poor passivation of Cd^2+^ may be attributed to its high solubility and strong reactivity, which results in a passivation process that is more complex and requires more intricate reaction pathways. Additionally, the lower affinity of Cd^2+^ for nZVI particles may result in slower adsorption and reaction rates on the surface of nZVI, ultimately affecting the passivation efficiency.^42^

Additionally, the immobilization efficiency of these agents was evaluated at different initial concentrations of Pb^2^+, Cd^2^+, and AsO_4_^3−^ (ranging from 10 mg L^−1^ to 500 mg L^−1^), and consistent trends in immobilization across the varying concentrations were observed. These experimental results demonstrate that the as-prepared nZVI is a promising passivation agent for removing the heavy metals AsO_4_^3−^, Pb^2+^, and Cd^2+^ from soil. The 99.99% passivation rate for AsO_4_^3−^ falls within the detection range of ICP-AES (LOD ≈ 0.005 mg L^−1^), and consistent results across variable groups support its validity.

**Fig. 4:**
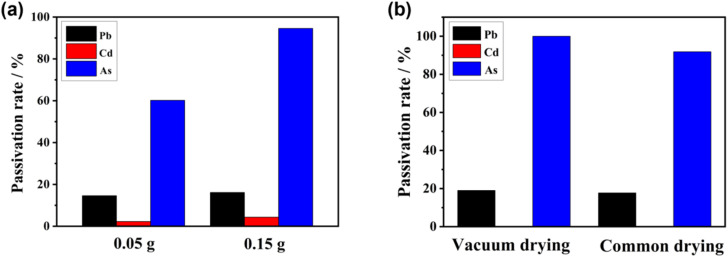
Relationship among the passivation rates of Pb^2+^, Cd^2+^, and AsO_4_^3−^ in soil and (a) the dosage of nZVI passivation agent as well as (b) drying conditions.

We further investigated the capacity of nZVI samples prepared under different drying conditions to adsorb AsO_4_^3−^, Pb^2+^, and Cd^2+^ in soil. Fig. 4a shows that the capacity of the nZVI passivation agents to adsorb the tested heavy metal ions in the soil is ranked as AsO_4_^3−^ > Pb^2+^ > Cd^2+^, and at a dosage of 0.15 g, it provides the passivation rates of 16.17%, 2.02%, and 94.56% for Pb^2+^, Cd^2+^, and AsO_4_^3−^, respectively. This demonstrates that AsO_4_^3−^ is preferentially adsorbed by nZVI, which occurs because Pb, Cd, and As exhibit different redox potentials (*E*_0h_), reflecting their different removal mechanisms upon contact with nZVI.^38^ The standard *E*_0h_ for Pb^2+^ (−0.13 V) is somewhat higher than that of Fe^2+^/Fe (−0.44 V), which corresponds to the moderate reduction in the adsorption capacity for Pb^2+^.^43^ In contrast, the standard *E*_0h_ for Cd^2+^ is −0.40 V, which is similar to that for Fe^2+^/Fe. Thus, Cd^2+^ mainly undergoes adsorption and precipitation during the adsorption process.

Additionally, the standard *E*_0h_ for As(v) is −0.56 V, and that for As(iii) is −0.18 V, which demonstrates that As(iii) can be easily oxidized to As(v) by nZVI. This enables more efficient adsorption and passivation of AsO_4_^3−^ through multi-layer reactions with nZVI, accompanied by co-precipitation.^44^ Although the current study focuses on representative contaminated soil samples, previous reports have demonstrated that nZVI performs effectively across a variety of soil types, including acidic, alkaline, and organic-rich soils. For instance, Kanel *et al.* reported successful arsenic removal in subsurface soils using nZVI,^45^ Fang *et al.* confirmed stable Pb^2+^ and Cd^2+^ immobilization in soils of varied textures,^46^ and Wang *et al.* discussed the reactivity and stability of nZVI under different pH and organic matter conditions.^47^ These findings support the broader applicability of nZVI in diverse remediation contexts.

### 3.3 Analysis of the passivation mechanism

**Fig. 5:**
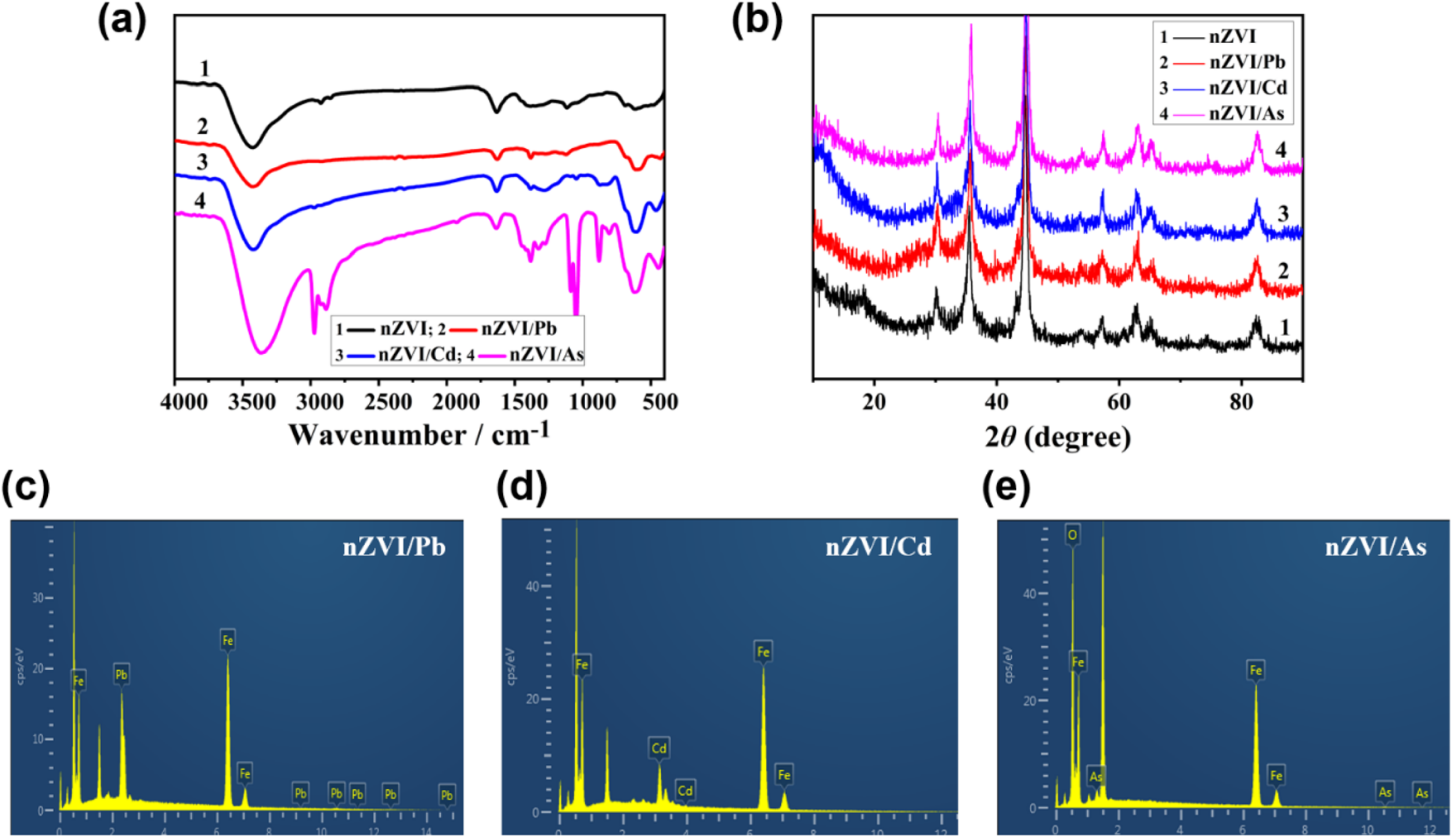
(a) FT-IR spectra, (b) XRD patterns, and EDS element composition of nZVI samples after adsorption of different heavy metals: (c) nZVI/Pb, (d) nZVI/Cd, and (e) nZVI/As.

To further verify the interaction mechanism of nZVI with Pb^2+^, Cd^2+^, and AsO_4_^3−^, we conducted FT-IR, XRD, and EDS analyses of nZVI/M (M represents Pb, Cd, and As; *i.e.*, Pb^2+^, Cd^2+^, and AsO_4_^3−^). As shown in Fig. 5a, nZVI exhibited four characteristic absorption bands at 3419, 1644, 1336, 1061, and 520 cm^−1^.^48^ The absorption band at 3419 cm^−1^ was assigned to O–H of iron oxide, and that at 1336 cm^−1^ was ascribed to Fe_2_O_3_, Fe_3_O_4_, and FeOOH, which are commonly formed on the oxidized shell of zero-valent iron (Fe^0^). While FT-IR does not directly detect metallic Fe^0^, the presence of these species may serve as indirect evidence of its existence. In addition, nZVI does not show strong absorption bands below 900 cm^−1^, indicating its mild oxidation.^49^ This mild oxidation of nZVI may be further influenced by the interaction with Pb^2+^, Cd^2+^, and AsO_4_^3−^, as seen in the changes in these characteristic bands.^50^

Notably, nZVI retained its composition after adsorbing the tested heavy metal ions. Fig. 5b shows the XRD patterns of nZVI and nZVI/M. The major XRD peaks at 2*θ* of 44.8°, 65.2°, and 82.5° correspond to the (110), (200), and (211) crystal faces of cubic Fe (PDF #87-0722), respectively,^51^ and the XRD signals of the (400), (422), (440), (620), and (551) facets of cubic ferric oxide (PDF 88-0866) emerge at 2*θ* of 43.1°, 53.5°, 62.5°, 71.0°, and 82.0°, respectively.^52^ This indicates that there is an oxide layer on the surface of nZVI. The high similarity among the XRD signals of nZVI and nZVI/M demonstrates that nZVI retains its crystal structure after adsorbing the tested heavy metals.

Fig. 5c–e and Table 1 show the EDS analysis results for samples nZVI/Pb, nZVI/Cd, and nZVI/As. The magnification used in the EDS images (c–e) was 20*k*×. Sample nZVI/Pb mainly consists of Fe (70.7%) and Pb (29.3%), which corresponds to the strong complexing reaction between nZVI and Pb^2+^. Similarly, Fe (86.1%) and Cd (13.9%) are the major elements in sample nZVI/Cd, indicating a chemical interaction between nZVI and Cd. In addition, as seen in Fig. 5e, sample nZVI/As mainly comprises Fe (mass fraction 74.8%), As (4.2%), and O (21.0%). The oxygen signal is reasonably attributed to adsorbed arsenate (AsO_4_^3−^) species, which inherently contain four oxygen atoms per ion. As no other oxygen-containing reagents were used and the sample was thoroughly rinsed before analysis, it is likely that the detected O originates from the AsO_4_^3−^ adsorbed on the nZVI surface. Thus, nZVI can passivate Pb^2+^, Cd^2+^, and AsO_4_^3−^ through adsorption, co-precipitation, and chemical reaction.

**Table 1:** EDS element composition of nZVI/M

Passivation agents	Element composition (wt%)				
	Fe	Pb	Cd	As	O
nZVI/Pb	70.7	29.3	—	—	—
nZVI/Cd	86.1	—	13.9	—	—
nZVI/As	74.8	—	—	4.2	21.0

To further elucidate the redox transformation of Fe^0^ and its involvement in the passivation process, advanced characterization techniques such as X-ray photoelectron spectroscopy (XPS) or Mössbauer spectroscopy are highly recommended. Although these analyses were not conducted in this study due to instrumental limitations, the conversion of Fe^0^ to Fe^3+^ was indirectly evidenced by the FT-IR, XRD, and EDS results, which indicate the formation of surface oxide layers. These oxides likely participate in the formation of Fe–O–M (M = Pb, Cd, As) coordination complexes, thereby contributing to the immobilization of heavy metals. In future work, XPS will be introduced to directly characterize the valence states of Fe and further clarify the passivation mechanism. Previous studies have confirmed the effectiveness of XPS in analyzing the oxidation states and surface interactions of Fe-based materials.^53–55^

**Fig. 6:**
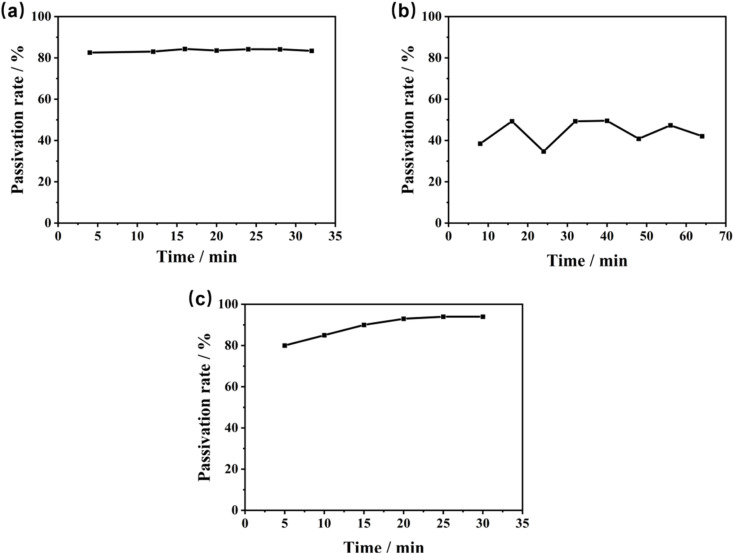
Variation of the passivation rates of Pb^2+^, Cd^2+^, and AsO_4_^3−^ with time: (a) Pb^2+^, (b) Cd^2+^, and (c) AsO_4_^3−^.

Fig. 6 shows the variation of the passivation rate of Pb^2+^, Cd^2+^, and AsO_4_^3−^ ions with time. The passivation rates of AsO_4_^3−^ and Pb^2+^ are higher than those of Cd^2+^. The passivation rate of Pb^2+^ slightly varied with extended adsorption time until it reached adsorption equilibrium in approximately 5 min, corresponding to rapid adsorption and passivation. The passivation rate for Cd^2+^ fluctuated within a certain range and leveled off in approximately 15 min. Additionally, the passivation rate for AsO_4_^3−^ approached 100% in the whole adsorption experiment. These results indicate that the prepared nZVI passivation agent can rapidly react with Pb^2+^, Cd^2+^, and AsO_4_^3−^ ions in the soil, thereby achieving their efficient passivation.

**Fig. 7:**
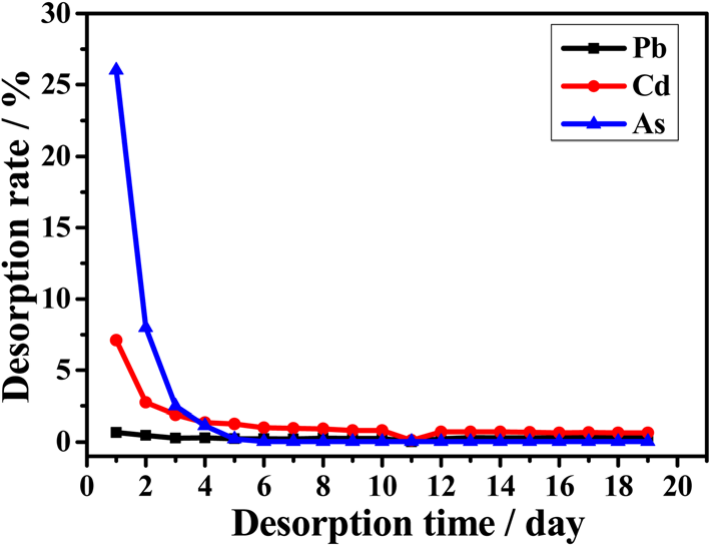
Relationship between the desorption rate of nZVI/M and desorption time.

Furthermore, we conducted 19 consecutive days of desorption experiments to evaluate the adsorption–desorption behaviour of Pb^2+^, Cd^2+^, and AsO_4_^3−^ ions. Fig. 7 shows the relationship between the desorption rate of nZVI/M and desorption time. On the first day of desorption, nZVI/Pb exhibited the lowest desorption rate of 0.7% and then quickly reached adsorption–desorption equilibrium, indicating its strong stability. Additionally, the desorption rates of nZVI/Cd and nZVI/As were relatively high, at 7.1% and 26.0%, respectively, on the first day of desorption and then reached adsorption–desorption equilibrium on day 6, indicating that they are less stable than nZVI/Pb, as discussed earlier.

Table 2 presents the adsorption details of Pb^2+^, Cd^2+^, and AsO_4_^3−^ by nZVI. The physical adsorption of Pb^2+^ and Cd^2+^ by nZVI is 1.7% and 5.6%, respectively, which are lower than that of AsO_4_^3−^ (29.3%), while the chemisorption percentages for Pb^2+^, Cd^2+^, and AsO_4_^3−^ are 93.0%, 74.8%, and 32.9%, respectively. This is in accordance with the desorption rates for nZVI/Pb, nZVI/Cd, and nZVI/As and is consistent with the stability of the adsorbed complexes as well (Fig. 7).

**Table 2:** The data for Pb^2+^, Cd^2+^, and AsO_4_^3−^ from the nineteen-day continuous desorption experiment

Adsorbate	Adsorption–desorption percentage (%)			Adsorption capacity (mg g^−1^)
	Desorption	Physical adsorption	Chemical adsorption	
Pb^2+^	5.3	1.7	93.0	146.7
Cd^2+^	19.6	5.6	74.8	67.1
AsO_4_^3-^	37.8	29.3	32.9	13.0

**Fig. 8:**
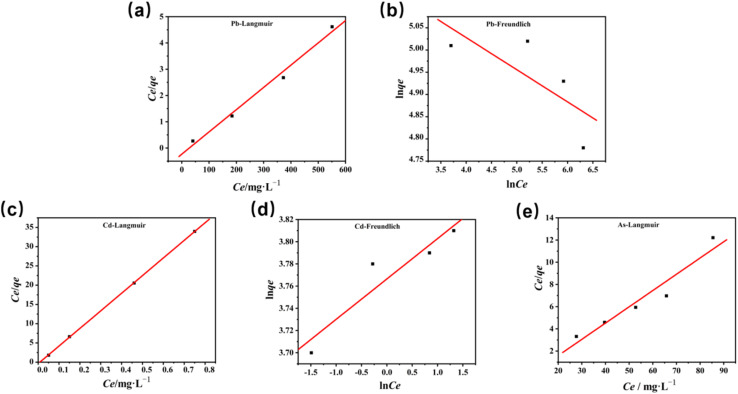
Langmuir and Freundlich adsorption isotherms of (a and b) Pb^2+^, (c and d) Cd^2+^, and (e) AsO_4_^3−^ upon being adsorbed by nZVI.

To explore the maximum adsorption capacity of nZVI for heavy metal ions Pb^2+^, Cd^2+^, and AsO_4_^3−^, we fitted the adsorption isotherms with the experimental data. For that purpose, Langmuir, Freundlich, and Dubinin–Radushkevich (D–R) curves are widely used.^36^ Herein, we adopted Langmuir (eqn (1)) and Freundlich (eqn (2)) adsorption isotherms to determine the maximum adsorption capacity of nZVI for Pb^2+^, Cd^2+^, and AsO_4_^3−^:1
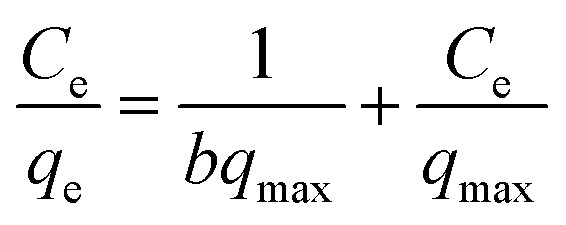
2
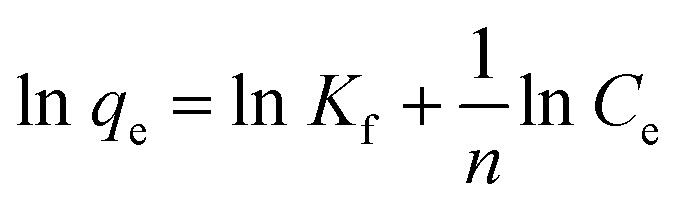
where *C*_e_ denotes the equilibrium concentration (mg L^−1^); *q*_e_ denotes the equilibrium adsorption capacity (mg g^−1^); *b* denotes the Langmuir adsorption equilibrium constant (L mg^−1^); *q*_max_ denotes the maximum adsorption capacity (mg g^−1^); *K*_f_ denotes the Freundlich affinity coefficient (mg^1−1/*n*^ L^1/*n*^ g^−1^); and *n* denotes the Freundlich constant. Fig. 8 shows the adsorption isotherms of Pb^2+^, Cd^2+^, and AsO_4_^3−^ upon being adsorbed by nZVI, and Table 3 lists the fitted parameters of the Langmuir and the Freundlich adsorption isotherm equations. The linear correlation coefficients (*R*^2^) for Pb^2+^ are 0.99 (Langmuir adsorption model) and 0.58 (Freundlich adsorption model), respectively, which indicates that the adsorption of Pb^2+^ by nZVI follows the Langmuir adsorption model, which is monolayer adsorption. The *R*^2^ for Cd^2+^ is 0.99 (Langmuir adsorption model) and 0.88 (Freundlich adsorption model), which indicates that the adsorption of Cd^2+^ by nZVI better conforms to the Langmuir adsorption model, which describes monolayer adsorption.

**Table 3:** Langmuir and Freundlich adsorption isotherm parameters for Pb^2+^, Cd^2+^, and AsO_4_^3−^

Ion species	Adsorption isotherm parameters					
	Langmuir model			Freundlich model		
	*b* (L mg^−1^)	*q*_max_ (mg g^−1^)	*R* ^2^	*K*_f_ (mg^1−1/*n*^ L^1/n^ g^−1^)	1/*n*	*R* ^2^
Pb^2+^	−0.04	117.65	0.99	442.53	−0.26	0.58
Cd^2+^	3.49	45.45	0.99	40.03	0.04	0.88
AsO_4_^3-^	−0.11	6.82	0.93	11.78	−0.09	0.11

Similarly, the linear correlation coefficient for AsO_4_^3−^, simulated with the Langmuir adsorption model, is 0.93, which is much greater than that simulated with the Freundlich model (0.11). The agreement with the Langmuir model indicates that the adsorption of AsO_4_^3−^ by nZVI is consistent with the Langmuir model, which describes monolayer adsorption. Although the Freundlich model fitting in some cases suggests possible surface heterogeneity that may influence the adsorption behavior, the Langmuir model still provides the most optimal fit, confirming the single-layer adsorption nature of the process for all tested ions. These findings help explain the observed differences in passivation performance, especially for AsO_4_^3−^, which exhibited a higher affinity for nZVI. In addition, the maximum adsorption capacities of nZVI for Pb^2+^, Cd^2+^, and AsO_4_^3−^ are 117.65 mg g^−1^, 45.45 mg g^−1^, and 6.82 mg g^−1^, respectively, indicating that the saturated adsorption capacity of nZVI for the tested heavy metal ions follows the order of Pb^2+^ > Cd^2+^ > AsO_4_^3−^.

**Fig. 9:**
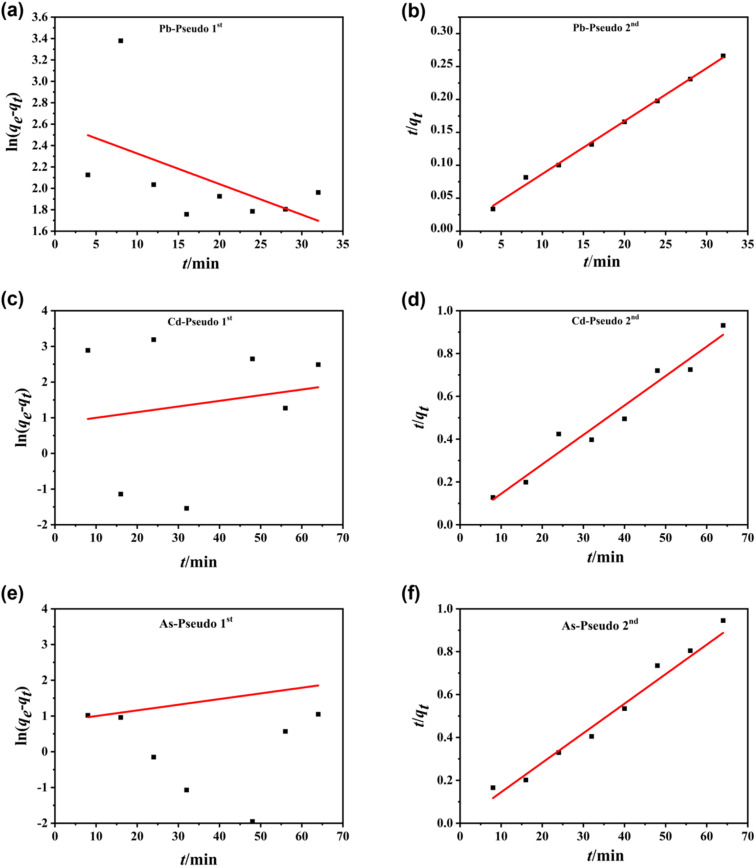
Pseudo 1st-order and pseudo 2nd-order kinetic curves of adsorption for (a and b) Pb^2+^, (c and d) Cd^2+^, and (e and f) AsO_4_^3−^ by nZVI.

Furthermore, we adopted quasi-first-order kinetics (eqn (3)) and quasi-second-order kinetics (eqn (4)) models to simulate the adsorption kinetics of Pb^2+^, Cd^2+^, and AsO_4_^3−^ on nZVI:3
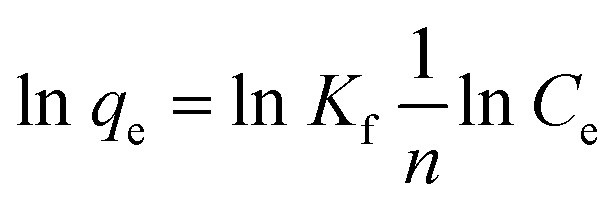
4
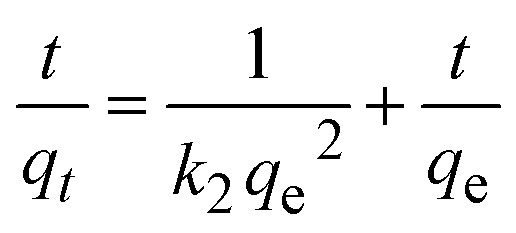
where *q*_e_ denotes the equilibrium adsorption amount (mg g^−1^), *q*_t_ denotes the adsorption amount at time *t* (mg g^−1^), *k*_1_ denotes the adsorption rate constant of the quasi-first-order kinetics model (min^−1^), and *k*_2_ denotes the adsorption rate constant of the quasi-second-order kinetics model (mg g^−1^ min^−1^). The adsorption curves of Pb^2+^, Cd^2+^, and AsO_4_^3−^ over nZVI simulated with eqn (3) and (4) are shown in Fig. 9, and the corresponding specific adsorption parameters are presented in Table 4. When the initial concentration of Pb^2+^ is 720.80 mg L^−1^, the linear correlation coefficients (*R*^2^) simulated with eqn (3) and (4) are 0.28 and 0.99, respectively, which indicates that the adsorption of Pb^2+^ ions by nZVI is consistent with the pseudo-second-order kinetic model. Similarly, when the initial concentration of Cd^2+^ is 817.00 mg L^−1^, the linear correlation coefficient simulated with the pseudo-first-order kinetic model is only 0.03, much lower than that simulated with the pseudo-second-order kinetic model (0.96). This indicates that the adsorption of Cd^2+^ on nZVI also follows the pseudo-second-order kinetic model. In the case of AsO_4_^3−^, when the initial concentration is 972.00 mg L^−1^, the linear correlation coefficients of the pseudo-first-order and pseudo-second-order kinetic models are 0.02 and 0.97, respectively. This demonstrates that the adsorption of AsO_4_^3−^ by nZVI also follows the pseudo-second-order kinetic model, similar to the cases for Pb^2+^ and Cd^2+^. Thus, the adsorption of Pb^2+^, Cd^2+^, and AsO_4_^3−^ by nZVI is dominated by chemisorption, and the adsorption process is governed by the pseudo-second-order kinetic model.

**Table 4:** Pseudo 1st-order and pseudo 2nd-order kinetic parameters for Pb^2+^, Cd^2+^, and AsO_4_^3−^ adsorption by nZVI

Ion species	*C*_0_ (mg L^−1^)	Pseudo-first-order kinetics			Pseudo-second-order kinetics		
		*k*_1_ (min^−1^)	*q*_e_ (mg g^−1^)	*R* ^2^	*k*_2_ (mg g^−1^ min^−1^)	*q*_e_ (mg g^−1^)	*R* ^2^
Pb^2+^	720.80	0.03	13.62	0.28	0.01	125.00	0.99
Cd^2+^	817.00	−0.02	2.32	0.03	0.03	72.46	0.96
AsO_4_^3-^	972.00	0.02	0.75	0.02	0.02	14.29	0.97

### 3.4 Reusability performance of nZVI

**Fig. 10:**
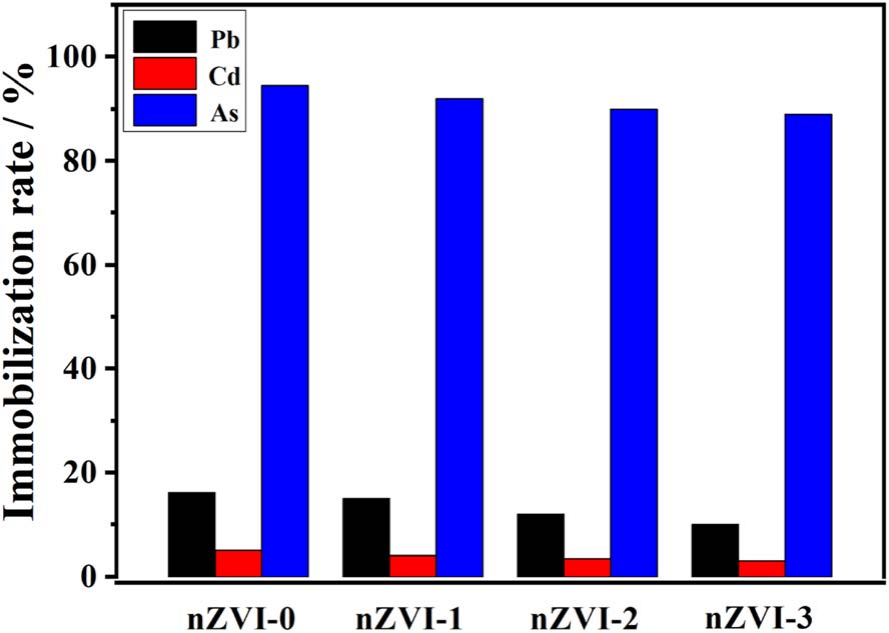
Immobilization rate of Pb^2+^, Cd^2+^, and AsO_4_^3−^ by nZVI after repeated reuse cycles (nZVI-0 to nZVI-3).

As shown in Fig. 10, the efficiency of nZVI in immobilizing AsO_4_^3−^ remained consistently above 90% after three reuse cycles (nZVI-1 to nZVI-3), indicating the high stability and redox durability of the material. In contrast, the immobilization rates for Pb^2+^ and Cd^2+^ exhibited slight decreases with repeated use, but continued to retain more than 80% and 70% of the initial capacity, respectively. These results suggest that nZVI can maintain its effective performance during multiple remediation cycles, particularly for anionic contaminants such as AsO_4_^3−^. The moderate decline in Pb^2+^ and Cd^2+^ immobilization could be attributed to partial surface oxidation or saturation of active binding sites. Overall, this highlights the economic and practical feasibility of nZVI as a recyclable material for heavy metal remediation.

### 3.5 Comparison of nZVI with conventional remediation materials

**Fig. 11:**
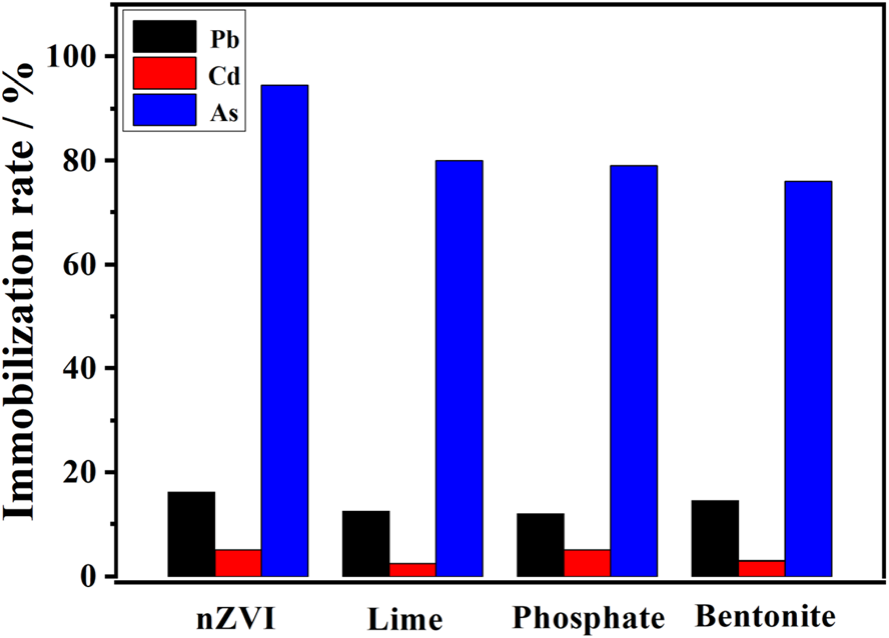
Relationship between the immobilization rate for Pb^2+^, Cd^2+^, and AsO_4_^3−^ and different remediation materials (nZVI, lime, phosphate, and bentonite).

To evaluate the remediation efficiency of nZVI, a comparative experiment was conducted using lime, phosphate, and bentonite as reference materials. As shown in Fig. 11, all materials exhibited varying degrees of immobilization on Pb^2+^, Cd^2+^, and AsO_4_^3−^. nZVI immobilized AsO_4_^3−^ at the highest rate (over 90%), indicating strong redox and adsorption ability.

In addition, nZVI exhibited a comparable or slightly better performance than the other materials in stabilizing Pb^2+^ and Cd^2+^. This can be attributed to the large specific surface area and reactive Fe^0^/Fe^3+^ species of nZVI. Thus, nZVI showed superior performance in treating cationic and anionic heavy metals, highlighting its potential as a versatile soil remediation agent.

### 3.6 Schematic illustration of nZVI synthesis and its passivation process towards Pb^2+^, Cd^2+^ and AsO_4_^3-^

**Fig. 12:**
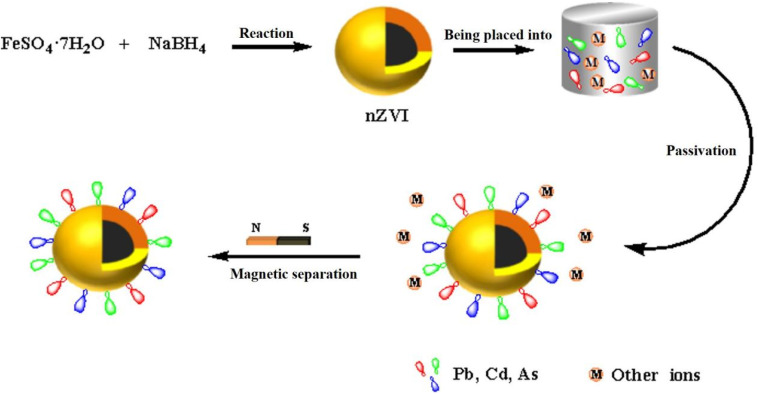
Schematic diagram for the preparation of nZVI and the passivation process for Pb^2+^, Cd^2+^, and AsO_4_^3−^.

Fig. 12 shows a schematic diagram for the synthesis of nZVI and its passivation of Pb^2+^, Cd^2+^, and AsO_4_^3−^. With FeSO_4_·7H_2_O as the iron source and NaBH_4_ as the reductant, nZVI is obtained by a one-step liquid-phase chemical route. The as-prepared nZVI is a core–shell structure consisting of a zero-valent iron core and a thin amorphous shell of Fe(ii)/Fe(iii) oxides.^20,21^ It can passivate Pb^2+^, Cd^2+^, and AsO_4_^3−^ ions *via* adsorption, co-precipitation, and chemical interactions. Furthermore, nZVI/M can be easily collected by applying an external magnetic field, which is favorable for the recycling of the nano-adsorbent.

## 4. Conclusions

nZVI was prepared by a one-pot liquid-phase chemical method, and optimum synthesis conditions for nZVI were established. It was found that the as-prepared nZVI can be directly used to efficiently passivate Pb^2+^, Cd^2+^, and AsO_4_^3−^ ions in contaminated soil, and its ability to adsorb the target heavy metal ions is governed by drying conditions and NaBH_4_ dosage. The adsorption of the tested heavy metal ions by nZVI follows a Langmuir isotherm model and pseudo-second-order kinetics equation, and therefore is characterized by saturated monolayer adsorption dominated by chemical interaction. The difference in the saturated adsorption capacity of nZVI for the tested heavy metal ions is related to their different chemisorption percentages. In summary, our data indicate that the as-synthesized nZVI is a promising high-performance nano-adsorbent for remediating heavy metal anion–cation co-contaminated soil.

## Conflicts of interest

There are no conflicts to declare.

## Data Availability

The data are available on request from the authors. The data that support the findings of this study are available from the corresponding author upon reasonable request.

## References

[cit1] Saini K., Singh J., Malik S. (2024). *et al.*, Metal-Organic Frameworks: A promising solution for efficient removal of heavy metal ions and organic pollutants from industrial wastewater. J. Mol. Liq..

[cit2] Molla A. H., Saha R., Sultana S. (2025). *et al.*, Assessment of toxicities and threat to biodiversity in an industrial effluent discharged environment. Int. J. Environ. Sci. Technol..

[cit3] Jobby R., Jha P., Yadav A. K. (2018). *et al.*, Biosorption and biotransformation of hexavalent chromium [Cr(VI)]: A comprehensive review. Chemosphere.

[cit4] Liu L., Li W., Song W. (2018). *et al.*, Remediation techniques for heavy metal-contaminated soils: Principles and applicability. Sci. Total Environ..

[cit5] Vodyanitskii Y. N. (2013). *et al.*, Contamination of soils with heavy metals and metalloids and its; ecological hazard (analytic review). Eurasian Soil Sci..

[cit6] Bi X., Shan M., Zou X. (2024). *et al.*, Study of adsorption capacity and mechanism of nanoalumina for arsenic Ion by isothermal adsorption model simulations. Environ. Technol. Innovation.

[cit7] Morgan S. E., DeLouise L. A., Zhang Y. (2024). *et al.*, Assessing bioactivity of environmental water samples filtered using nanomembrane technology and mammalian cell lines. Eco-Environ. Health.

[cit8] Petit J. C. J., Maggi P., Pirard C. (2022). *et al.*, Human biomonitoring survey (Pb, Cd, As, Cu, Zn, Mo) for urban gardeners exposed to metal contaminated soils. Environ. Pollut..

[cit9] Gao M., Zhao X., Zou X. (2023). *et al.*, Preparation of fluorescently and biologically active chain-like chitosan nanocomposite and its use in separating MBP-tagged proteins and as fluorescent tracer of tobacco. Sens. Actuators, B.

[cit10] Gao M., Liu Q., Zou X. (2022). *et al.*, Facile synthesis of peanut-like Sn-doped silica nano-adsorbent for affinity separation of proteins. RSC Adv..

[cit11] Huang J., Yang X., Zhang X. (2019). *et al.*, Arsenic contamination in groundwater and its health impacts in rural areas: A case study in China. Environ. Toxicol. Pharmacol..

[cit12] Gao M., Li L., Zou X. (2022). *et al.*, Synthesis of multifunctional silica composite nanospheres and their application in separation of MBP-tagged protein. Mater. Lett..

[cit13] Yao Z. T., Li J. H., Xie H. H. (2012). *et al.*, Review on remediation technologies of soil contaminated by heavy metals. Procedia Environ. Sci..

[cit14] Li Z., Zhang J., Wang L. (2020). *et al.*, Environmental policies and their impact on heavy metal pollution in China: A review. Sci. Total Environ..

[cit15] Yu S., Tan Z., Lai Y. (2023). *et al.*, Nanoparticulate pollutants in the environment: Analytical methods, formation, and transformation. Eco-Environ. Health.

[cit16] Smith E., Thavamani P., Ramadass K. (2015). *et al.*, Remediation trials for hydrocarbon-contaminated soils in arid environments: evaluation of bioslurry and biopiling techniques. Int. Biodeterior. Biodegrad..

[cit17] Liang J., Han Lu, Zou X. (2021). *et al.*, Fast and efficient immobilization behavior of bifunctional magnetic nano-amendment against multi-heavy metal. Chin. J. Inorg. Chem..

[cit18] Khaligh N. G., Johan M. R., Lee S. H., Johan M. R. (2018). *et al.*, Recent application of the various nanomaterials and nanocatalysts for the heavy metals' removal from wastewater. Nano.

[cit19] Zou X. Y., Zhao Y. B., Zhang Z. J. (2020). *et al.*, A novel method to prepare hydroxyapatite nanotubes and its immobilization activities against heavy metal ions in solutions. Chin. J. Inorg. Chem..

[cit20] Zou X. Y., Zhao Y. B., Zhang Z. J. (2019). *et al.*, Preparation of hydroxyapatite nanostructures with different morphologiesand adsorption behavior on seven heavy metals ions. J. Contam.
Hydrol..

[cit21] Tao L., Mi X., Ren H. (2022). *et al.*, Stabilization of heavy metals in mining soil using palygorskite loaded by nanoscale zero-valent iron. Int. J. Environ. Sci. Technol..

[cit22] Singh S., Kapoor D., Khasnabis S. (2021). *et al.*, Mechanism and kinetics of adsorption and removal of heavy metals from wastewater using nanomaterials. Environ. Chem. Lett..

[cit23] Matos M. P. S. R., Correia A. A. S., Rasteiro M. G. (2017). *et al.*, Application of carbon nanotubes to immobilize heavy metals in contaminated soils. J. Nanopart. Res..

[cit24] Limin W., Wang P., Yin Z. (2024). *et al.*, ZmHAK17 encodes a Na+-selective transporter that promotes maize seed germination under salt conditions. New Crops.

[cit25] Mohamadiun M., Dahrazma B., Saghravani S. F. (2018). *et al.*, Removal of cadmium from contaminated soil using iron (III) oxide nanoparticles stabilized with polyacrylic acid. J. Environ. Eng. Landsc. Manag..

[cit26] Bin Q., Lin B., Zhu K. (2020). *et al.*, Superior trichloroethylene removal from water by sulfide-modified nanoscale zero-valent iron/graphene aerogel composite. J. Environ. Sci..

[cit27] Wang C. M., Baer D. R., Amonette J. E. (2009). *et al.*, Morphology and electronic structure of the oxide shell on the surface of iron nanoparticles. J. Am. Chem. Soc..

[cit28] Jifu Li, Jing T., Zhou M. (2024). *et al.*, Research progress on the physiological and molecular mechanisms underlying soybean aluminum resistance. New Crops.

[cit29] Cao Z., Xu J., Li H. (2020). *et al.*, Dechlorination and defluorination capability of sulfidized nanoscale zerovalent iron with suppressed water reactivity. Chem. Eng. J..

[cit30] Yang Y., Zhang Z., Wan M. (2020). *et al.*, A facile method for the fabrication of silver nanoparticles surface decorated polyvinyl alcohol electrospun nanofibers and controllable antibacterial activities. Polymers.

[cit31] Ni Z., Gu X., He Y. (2018). *et al.*, Synthesis of silver nanoparticle-decorated hydroxyapatite (HA@ Ag) poriferous nanocomposites and the study of their antibacterial activities. RSC Adv..

[cit32] Gao M., Xu C., Zou X. (2024). *et al.*, Magnetically responsive ferriferrous oxide@chitosan-polyacrylic acid (or dextrin) nanocomposites for highly efficient separation and purification of targeted fusion proteins. Chem. Eng. J..

[cit33] Chen M., Xu H., Zhang Y. (2022). *et al.*, Effective removal of heavy metal ions by attapulgite supported sulfidized nanoscale zerovalent iron from aqueous solution. Colloids Surf., A.

[cit34] Galdames A., Ruiz-Rubio L., Orueta M. (2020). *et al.*, Zero-valent iron nanoparticles for soil and groundwater remediation. Int. J. Environ. Res. Public Health.

[cit35] Garcia A. N., Boparai H. K., Chowdhury A. I. A. (2020). *et al.*, Sulfidated nano zerovalent iron (S-nZVI) for *in situ* treatment of chlorinated solvents: a field study. Water Res..

[cit36] Turabik M., Simsek U. B. (2017). Effect of synthesis parameters on the particle size of the zero valent iron particles. Inorg. Nano-Met. Chem..

[cit37] Li X. Q., Zhang W. X. (2007). Sequestration of metal cations with zerovalent iron nanoparticles-A study with high resolution X-ray photoelectron spectroscopy (HR-XPS). J. Phys. Chem. C.

[cit38] Lei Z., Song X., Ma G. (2023). *et al.*, A review of recent studies on nano zero-valent iron activated persulfate advanced oxidation technology for the degradation of organic pollutants. New J. Chem..

[cit39] Huang P. P., Ye Z. F., Xie W. M. (2013). *et al.*, Rapid magnetic removal of aqueous heavy metals and their relevant mechanisms using nanoscale zero valent iron (nZVI) particles. Water Res..

[cit40] Yang Y., Zhang Z., Wan M. (2020). *et al.*, A facile method for the fabrication of silver nanoparticles surface decorated polyvinyl alcohol electrospun nanofibers and controllable antibacterial activities. Polymers.

[cit41] Zhou Y., Wang X., Zhang Y. (2024). *et al.*, Environmental remediation approaches by nanoscale zero-valent iron (nZVI) based on its reductivity. RSC Adv..

[cit42] Li X., Wu Z., Zhang W. (2022). *et al.*, The Effect of Nano Zero-Valent Iron on the Passivation of Heavy Metals in Soil: A Case Study of Cd^2+^. Environ. Sci. Technol..

[cit43] Lu D., Wang L., Zhang H. (2023). *et al.*, Removal of heavy metals in water using nano zero-valent iron composites: A review. J. Water Proc. Eng..

[cit44] Li Z., Wang L., Meng J. (2017). *et al.*, Zeolite-supported nanoscale zero-valent iron: New findings on simultaneous adsorption of Cd(II), Pb(II), and As(III) in aqueous solution and soil. J. Hazard. Mater..

[cit45] Alazaiza M. Y. D., Shadi M., Shanableh A. (2022). *et al.*, Nanoscale zero-valent iron application for the treatment of soil, wastewater and groundwater contaminated with heavy metals: a review. Desalin. Water Treat..

[cit46] Chen H., Qian L., Zhang Y. (2024). *et al.*, Performance of field demonstration nanoscale zero-valent iron in groundwater remediation: a review. Sci. Total Environ..

[cit47] Liu Y. Z., Wu W. M., Zeng R. J. (2022). *et al.*, Synergistic effect of soil organic matter and nanoscale zero-valent iron on biodechlorination. Environ. Sci. Technol..

[cit48] Goti M., Musi S., Mössbauer (2007). *et al.*, FT-IR and FE SEM investigation of iron oxides precipitated from FeSO_4_ solutions. J. Mol. Struct..

[cit49] Lu H., Qiao X., Wang W. (2015). *et al.*, Facile preparation of mesoporous silica/nano zero-valent iron composite for Pb(II) removal from aqueous solution. Desalin. Water Treat..

[cit50] Wang J., Li X., Zhang Y., Liu H. (2020). *et al.*, Effect of nZVI on the immobilization and passivation of Pb^2+^, Cd^2+^, and AsO_4_^3-^ in contaminated soil. Environ. Sci. Technol..

[cit51] Khurshid H., Mustafa M. R. U., Isa M. H. (2022). *et al.*, Adsorption of chromium, copper, lead and mercury ions from aqueous solution using bio and nano adsorbents: a review of recent trends in the application of AC, BC, nZVI and MXene. Environ. Res..

[cit52] Viotti P., Tatti F., Andrei F. (2022). *et al.*, Assessment of zerovalent iron nanoparticle (nZVI) efficiency for remediation of arsenic-contaminated groundwater: two laboratory experiments. Water.

[cit53] Liu T., Wang D., Chen Y. (2020). *et al.*, Insight into the removal mechanism of Pb(II) by nano zero-valent iron: A XPS and Mössbauer spectroscopy study. J. Hazard. Mater..

[cit54] Ezzatahmadi N., Ayoko G. A., Millar G. J. (2017). *et al.*, Clay-supported nanoscale zero-valent iron composite materials for the remediation of contaminated aqueous solutions: a review. Chem. Eng. J..

[cit55] Zhang L., Li Y., Wang X. (2024). *et al.*, Fabrication and antibacterial properties of electrospun nanofibers incorporating silver nanoparticles for environmental applications. Eco-Environ. Health.

